# Measuring communicative participation using the FOCUS^©^^1^: Focus on the Outcomes of Communication Under Six

**DOI:** 10.1111/cch.12049

**Published:** 2013-06-13

**Authors:** N Thomas-Stonell, K Washington, B Oddson, B Robertson, P Rosenbaum

**Affiliations:** * Bloorview Research Institute, Holland Bloorview Kids Rehabilitation HospitalToronto, ON, Canada; † University of TorontoToronto, ON, Canada; ‡ Department of Communication Sciences and Disorders, University of CincinnatiCincinnati, OH, USA; § School of Human Kinetics, Laurentian UniversitySudbury, ON, Canada; ¶ CanChild Centre for Childhood Disability Research, McMaster UniversityHamilton, ON, Canada

**Keywords:** communication, indicators, participation, preschool children, speech and language therapy

## Abstract

**Background:**

The FOCUS^©^ is a new outcome tool for use by both parents and clinicians that measures changes in the communicative participation skills of preschool children. Changes in communicative participation skills as measured by the FOCUS were compared across three groups of children: those with speech impairments only (SI), those with language impairments only (LI) and those with both speech and language impairments (S/LI).

**Methods:**

Participating families (*n* = 112, 75 male children) were recruited through 13 Canadian organizations. Children ranged from 10 months to 6 years 0 months (mean = 2.11 years; SD = 1.18 years) and attended speech-language intervention. Parents completed the FOCUS at the start and end of treatment. There were 23 children in the SI group, 62 children in the LI group and 27 children in the S/LI group. The average amount of the children's therapy varied from 7 to 10 h.

**Results:**

The FOCUS captures changes in communicative participation for children with a range of communication disorder types and severities. All three groups of children made clinically important improvements according to their FOCUS scores (MCID ≥ 16 points). The FOCUS captured improvements in intelligibility, independent communication, play and socialization.

**Conclusions:**

The FOCUS measured positive changes in communicative participation skills for all three groups of children after 7–10 h of speech-language therapy. An outcome measure that targets only specific speech and language skills would miss many of the important social function changes associated with speech-language treatment.

## Introduction

Children with speech and/or language impairments experience a multitude of participation restrictions and activity limitations as defined by the World Health Organizations' (WHO) International Classification of Functioning, Disability and Health – Children and Youth (ICF-CY). These restrictions extend beyond communication and include difficulties with reading, writing, spelling, focusing attention, thinking, calculating handling stress and forming adult–child, parent–child, and sibling relationships (McCormack *et al*. 2009, 2010). For some children with communication impairments, problematic social interactions and limited play skills can lead to peer rejection (Shepherd *et al*. 1994; Timler *et al*. 2005). By 3 years of age, these children may already be experiencing social isolation (Brinton & Fujiki 2005). Siblings report that they often protect their speech-impaired sibling from bullying by their peers (Barr *et al*. 2008; McCormack *et al*. 2009). Older children (6–11 years) with communication impairments expressed concerns about their academic achievement, friendships and standing out from the crowd (Owen *et al*. 2004). Early and effective speech-language therapy is critical to preventing these problems (Fujiki *et al*. 2001).

Measuring treatment outcomes is one way to improve services in an evidence-based manner, and inform clinical decision-making (Fujiki *et al*. 2001). Outcome measures help document the impact of intervention on children's lives (Gertner *et al*. 1994; Horowitz *et al*. 2006). Despite a move towards measuring the functional outcomes of intervention, however, few measures have been designed to capture broad communication-related outcomes such as quality of life and social participation (Kagan *et al*. 2008; Dempsey & Skarakis-Doyle 2010). This limits speech-language pathologists' knowledge about the potential changes in these skills following speech-language intervention (Thomas-Stonell *et al*. 2009; Dempsey & Skarakis-Doyle 2010; Washington 2010). To evaluate the full impact of intervention on a child's life, outcome measures must capture the spectrum of changes from individual deficits to life participation (Thomas-Stonell *et al*. 2009).

The FOCUS^©^ is a new outcome tool for use by either parents or clinicians. It consists of 50 items and takes 10 min to complete. Items were derived from a content analysis of 210 parents' observations of improvements in children's skills following speech-language therapy (Thomas-Stonell *et al*. 2009). Parents noted positive changes in speech, language, play, socialization, confidence and behaviour. The comments aligned with the WHO ICF-CY health framework, which provided a theoretical context for the development of the FOCUS (Thomas-Stonell *et al*. 2010).

The FOCUS was tested with 165 new families and revised according to parent and clinician feedback and item analysis. Items were selected for reliability and responsiveness. Item reduction resulted in an ever-increasing emphasis on Activities and Participation items. Over 90% of the items relate to the ICF-CY Activities and Participation domain (WHO 2007). FOCUS items are rated on 7-point Likert scales. There are two scales: one varies from ‘Not at all like my child’ to ‘Exactly like my child’; the second varies from ‘Cannot do at all’ to ‘Can always do without help’. Each item is scored from 1 to 7 resulting in a range of total scores from 50 to 350 points. Change is measured by comparing the total FOCUS score at the beginning and end of a treatment period. A higher change score indicate more change.

The ICF-CY defines ‘Activity’ as ‘the execution of a task or action by an individual’ and ‘Participation’ as ‘involvement in life situations’ (WHO 2007). The ICF-CY further distinguishes between the domains by using the qualifiers of ‘Capacity’ and ‘Performance’ with capacity defined as ‘an individual's optimal ability to execute a task of action in a standard environment’ and performance defined as ‘what an individual does in his current environment’, which includes a societal context (WHO 2007). The term ‘communicative participation’ has been defined as ‘communication in life situations where knowledge, information, ideas or feelings are exchanged’ (Eadie *et al*. 2006). The FOCUS captures changes in a child's capacity and performance as these relate to communication skills.

The FOCUS has demonstrated construct validity as a change-detecting instrument. As expected, the total score measured more change during a treatment period than during a wait list period. It demonstrated convergent validity with a health-related quality of life measure (Thomas-Stonell *et al*. 2010). It demonstrated convergent validity with the communication items of a measure of social/emotional skills and divergent validity with its non-communication related items (Thomas-Stonell *et al*. 2013). Inter-rater and test–retest reliability has also been established (Washington *et al*. 2007). The FOCUS has been able to measure changes in children's communicative participation skills after 9 h of speech-language therapy (Thomas-Stonell *et al*. 2010).

Because of the paucity of participation-level outcome measures, little is known about the impact of speech-language therapy on these skills (Thomas-Stonell *et al*. 2009; Dempsey & Skarakis-Doyle 2010; Washington 2010). If treatment reduces barriers to the child's participation, the associated improvement in participation skills may be similar for children with different communication disorders. It is possible, however, that improved participation skills differ for children who have different communication disorders and treatment goals. The objective of this study was to compare the communicative participation changes measured by the FOCUS in three groups of children who had different communication disorders: those with speech impairments only (SI), those with language impairments only (LI) and those with both speech and language impairments (S/LI).

## Participants

### Demographics

A convenience sample of 205 families was recruited from 13 partner organizations across Canada. Following ethical approval from each organization, speech-language pathologists approached parents/caregivers of children less than 6 years old to participate, following a standard recruiting script. Inclusion criteria included children who were identified by registered speech-language pathologists as having a communication disorder and recommended for intervention.

Forty families withdrew from speech-language therapy or transferred to another programme and 12 families withdrew from the study because of time commitments. There were missing data for 41 families. Complete data were obtained for 112 families. Demographics and variables related to speech-language treatment are described in Table 1. There were 23 children in the SI group, 62 children in the LI group and 27 children in the S/LI group. Communication disorder severity ratings in Table 1 used the Communication Function Classification System (CFCS), a valid and reliable five-level classification system from Level 1 (most functional) to Level 5 (least functional) (Hidecker *et al*. 2011).

**Table 1:** Characteristics of the SI, LI and S/LI samples

	SI sample (*n* = 23)	LI sample (*n* = 62)	S/LI sample (*n* = 27)
Age	Mean = 3.75	Mean = 2.4	Mean = 2.8
	SD = 0.78	SD = 0.78	SD = 0.92
	Range = 1.3–5.7	Range = 1.3–4.9	Range = 1.4–4.4
Sex	Male = 56%	Male = 61%	Male = 70%
	Female = 44%	Female = 39%	Female = 30%
CFCS	Level 1 = 17%	Level 1 = 5%	Level 1 = 7%
	Level 2 = 17%	Level 2 = 2%	Level 2 = 19%
	Level 3 = 35%	Level 3 = 7%	Level 3 = 19%
	Level 4 = 30%	Level 4 = 51%	Level 4 = 44%
	Level 5 = 0%	Level 5 = 35%	Level 5 = 11%
Medical diagnoses*	Percent of sample = 22%	Percent of sample = 61%	Percent of sample = 67%
	Cleft lip/palate = 13%	Dev. delay = 35%	Dev. delay = 33%
	Cerebral palsy = 9%	Cerebral palsy = 13%	Syndromes = 15%
	Syndromes = 4%	Hearing loss = 8%	Hearing loss = 11%
Amount of treatment (hours)	Mean = 8.7	Mean = 10.7	Mean = 7.6
	SD = 7.9	SD = 7.4	SD = 3.4
	Range = 3–36	Range = 4–36	Range = 1–14
Treatment type†	Individual = 64%	Individual = 48%	Individual = 56%
	Group = 45%	Group = 31%	Home programming = 33%
	Home programming = 14%	Home programming = 27%	Group = 14%
		Parent training = 10%	Parent training = 14%

* The top three medical diagnoses for each group are reported.

† For these categories, percentages add up to more than 100% because some participants have more than one treatment type.

SI, speech impairment only; LI, language impairment only; S/LI, both speech and language impairments; CFCS, Communication Function Classification System.

### Procedures

Parents completed the FOCUS at the start and completion of a block of speech-language treatment. Speech-language treatment was provided in accordance with accepted clinical practice at each partner organization. Treatment models, goals, and frequency were determined by the treating speech-language pathologist for each specific child. For 83% of children in the SI group, treatment goals addressed both articulation/phonology and intelligibility. For the LI group, treatment goals addressed expressive language (89%), receptive language (67%), and pragmatic language skills (55%) individually or in combination. For 78% of the S/LI group, treatment goals addressed articulation/phonology, intelligibility and expressive language skills. Treatment frequency varied from weekly sessions to one session every 2 months; however, consistent with current service delivery models, most children in all three groups received individual therapy once a week. (Note: This study was designed to assess the responsiveness of the FOCUS. It was not designed to evaluate the effectiveness of these treatments.) Additional information related to speech-language treatment is included in Table 1. An anova was conducted to compare the three groups at the start and end of treatment to see if they were statistically equivalent. Changes measured by the FOCUS were also compared for the three groups using an anova.

FOCUS change scores were evaluated to determine whether or not a ‘minimal clinically important difference’ (MCID) had occurred. A ‘minimal clinically important difference’ (MCID) is defined as minimal changes in the child's function that are considered to be important to both the clinician and parent (Iyer *et al*. 2003). At the end of treatment, parent and speech-language pathologists were independently asked to complete a questionnaire describing the changes they had observed during treatment and explaining why these changes were important. These comments were examined to determine whether or not functional improvements in communication skills had occurred. A change of ≥16 FOCUS points was established empirically as being a MCID, as the descriptive comments indicated greater than 95% agreement between speech-language pathologists and parents that important functional changes had occurred at this level (Thomas-Stonell *et al*. 2013).

To identify the types of communicative participation changes achieved by each group of children, a descriptive analysis of the FOCUS items was completed. The 10 items that measured the most change for each group of children (i.e. 20% of FOCUS items) were identified. These items were examined to provide insight into communicative participation skills had made the most change during treatment and determine if the changes were similar across the groups (see Table 2). Any items that showed negative change were also examined.

**Table 2:** A comparison of communicative participation changes measured by the FOCUS for the SI, LI and S/LI groups

**Top change FOCUS items for the SI group**		**ICF-CY component**

1.	My child speaks slowly when not understood.	Body Functions
2.	My child's speech is clear.	Body Functions
3.	My child uses correct grammar when speaking.	Activity/Capacity
4.	My child can communicate independently with adults who do not know my child well.	Performance
5.	My child is understood the first time when s/he is talking with other children.	Performance
6.	My child is understood the first time when talking with adults who do not know my child well.	Performance
7.	My child takes turns.	Activity/Capacity
8.	My child can communicate independently with other children.	Performance
9.	My child can communicate independently.	Activity/Capacity
10.	My child can tell adults who do not know my child well about past events.	Performance

**Top change FOCUS items for the LI group**		**ICF-CY Component**

1.	My child uses language to communicate new ideas.	Activity/Capacity
2.	My child speaks in complete sentences.	Activity/Capacity
3.	My child's speech is clear.	Body Functions
4.	My child can communicate independently with other children.	Performance
5.	My child can communicate effectively with other children.	Performance
6.	My child uses communication to solve problems.	Activity/Capacity
7.	My child can communicate effectively with adults who do not know my child well.	Performance
8.	My child can string words together.	Activity/Capacity
9.	My child talks while playing.	Performance
10.	My child can carry on a conversation with other children.	Performance

**Top change FOCUS items for the S/LI group**		**ICF-CY Component**

1.	My child can talk to other children about what s/he is doing.	Performance
2.	My child can communicate independently with other children.	Performance
3.	My child conveys her/his ideas with words.	Activity/Capacity
4.	My child is confident communicating with adults who do not know my child well.	Personal Factors &
5.	My child needs help to be understood by other children.	Performance
6.	My child talks a lot.	Activity/Capacity
7.	My child can communicate independently with adults who do not know my child well.	Performance
8.	My child waits for her/his turn to talk.	Activity/Capacity
9.	My child is willing to talk to others.	Personal Factors & Activity/Capacity
10.	My child will ask for things from adults s/he knows well.	Performance

SI, speech impairment only; LI, language impairment only; S/LI, both speech and language impairments; ICF-CY, International Classification of Functioning, Disability and Health – Children and Youth.

## Results

Analyses indicated that the three groups were not equivalent prior to treatment (*F*_2,109_ = 19.0, *P* < 0.01). The SI group differed from the LI and S/LI groups. The LI and S/LI groups were equivalent. The SI group had higher FOCUS scores than either the LI and S/LI groups. The average start of treatment FOCUS total score for the SI group was 247 points compared with 156 for the LI group and 177 points for the S/LI group. The higher FOCUS scores for the SI group are likely because of a combination of age and severity. Children in the SI group were older and had milder communication impairments (see Table 1). The median CFCS level for the SI group was 3 (Effective Sender and Receiver with familiar partners) compared with a median CFCS level of 4 for both the LI and S/LI groups (Inconsistent Sender and/or Receiver with familiar partners). In addition, fewer children in the SI group had a medical diagnosis associated with their communication impairments.

Group differences persisted at the end of treatment with the SI group continuing to have significantly higher FOCUS total scores. All three groups made statistically significant changes from the start to the end of treatment and averaged more than the 16 points required for a MCID (SI group 18.3, t = 3.4, *P* < 0.01; LI group 18.2, t = 5.2, *P* < 0.01; S/LI group 25.3, t = 3.2, *P* < 0.01). The SI and LI groups made an average change on the FOCUS of 18 points. The S/LI group made an average change of 25 points. This difference of 7 points is not statistically different, indicating that the three groups made equivalent amounts of change (*F* < 1). The average change across the three groups was 19.9 points (CI 14.08–25.66; t = 6.80, *P* < 0.001).

The 10 FOCUS items that measured the most treatment change for each group were selected to provide insight into the types of communicative participation changes that were occurring in each group (see Table 2). Two of the top 10 items for the SI group pertained to improvements in speech intelligibility (ICF-CY Body Functions). Three items measuring capacity indicated positive changes in such skills as grammar and turn taking. Five of the top 10 items were performance items indicating an improvement in the children's ability to communicate independently with other children and adults. Seven items showed negative change for this group. These items suggest that the children were more reluctant to talk and frustrated when communicating. Parents felt their children's communication skills limited both their independence and learning.

The top items for the LI group included one body function item, four capacity items and five performance items. The body function item related to improved speech clarity. The capacity items related to improvements in expressive language skills such as using longer/better sentences and using language to communicate ideas and solve problems. The performance items captured improvements in the children's abilities to socialize and carry on conversations with other children. For this group, there were two negative change items. These items indicated that parents felt their child's communication skills continued to limit their independence and that they had more difficulties changing activities.

The top items for the S/LI group included four capacity items and six performance items. The capacity items related to improvements in expressive language skills (i.e. sentence length and using language to convey new ideas and solve problems). The performance items for the S/LI group captured improvements in the children's abilities to communicate independently with unfamiliar adults and other children. This is the only group that had two personal factors items in the top 10 items. These items indicated that the children gained confidence and were more willing to talk to both familiar adults and children. There were two negative change items for this group. These items indicated that the children were continuing to have difficulties changing activities and responding to questions.

The profiles for the three groups are slightly different. The SI children made more change than the LI and S/LI groups on the first 15 items. These items pertained to improved speech and expressive language skills, intelligibility and independent communication skills. Although there was room for improvement, they showed the least improvement of the three groups on the last 16 FOCUS items which related to play skills and socialization with other children. The children in the LI group made consistent changes across most of the FOCUS items. The children in the S/LI group also made changes across items, but similar to the SI group, they made less change on the last 16 items (i.e. play and socialization) than the LI group.

## Discussion

The FOCUS captures changes in communicative participation for children with a range of communication disorder types and severities. All three groups made statistical and clinically significant treatment change as measured by the FOCUS. The amount of change measured by the FOCUS was similar across groups, despite the differences in communication disorder severity and child age between the three groups. This demonstrates the ability of the FOCUS to capture improvements for children with different ages and communication disorder severities.

The 10 FOCUS items that measured the most treatment change were different across the three groups.

This supports the construct validity of the FOCUS as it measures changes specific to the different communication disorders and severities presented by the three groups of children (Streiner & Norman 2008). The findings also suggest that different communication disorders have different impacts on the children's communicative participation skills. Of the top 10 items, only the item ‘My child can communicate independently with other children’ measured high change for all three groups.

Although the SI group improved their intelligibility and ability to communicate independently with other children and adults they made the least change on the last 16 items (i.e. Part 2 of the FOCUS). These items measure the amount of assistance and/or cuing required for the child to interact effectively, play and carry on conversations with other children and unfamiliar adults. The average pre-treatment score for these items (Part 2 of the FOCUS) was 5 out of 7 (range = 4–6) indicating that further improvements could have been captured by the FOCUS if they had occurred. Results suggest, however, that improvements in socialization and play skills do not automatically occur for children with speech impairments, despite improved intelligibility. Peer interactions have been well documented as an area of participation restriction for children with communication impairments; however, studies have not evaluated whether children with speech impairments experience more participation restrictions than children with impaired language skills (McCormack *et al*. 2009). It may be that speech impairments are more obvious to other children than language impairments and cause children with these difficulties to experience greater participation restrictions.

Although the SI children improved their speech intelligibility and independent communication skills, parents noted some increased frustration and reluctance to talk. Possibly therapy increased the children's awareness of their speech difficulties leading to some reluctance to communicate with their peers. Perhaps, the children were frustrated by increased expectations from both parents and speech-language pathologists to produce their target sounds more clearly and communicate independently. These changes were not represented in the parents' and speech-language pathologists' comments suggesting that they were not viewed as concerns. It is recommended, however, that parents and speech-language pathologists monitor frustration levels during the therapy process. Therapy may need to include an additional emphasis on social skills and play to help these children gain confidence, enter into play activities and interact better with their peers.

## Limitations

Study participants represented a convenience sample of children receiving speech-language therapy. It is difficult to know whether the profiles obtained in this study will generalize to the larger population of children with speech and/or language disorders. There are few measures that capture the communicative participation changes associated with speech-language therapy; therefore, little is known about the impact of speech and language treatment on these skills.

Further research is needed to determine how both positive and negative communicative participation changes are affected by different treatment goals and strategies (i.e. increased expectations place on the child to communicate). Parents have the greatest opportunities to observe their children's communication and socialization skills in a variety of environments. Therefore, they can be considered ‘gold standard’ observers. It is recommended that future research examine the agreement between parents' FOCUS scores and those of other observers such as teachers and early childhood educators.

## Conclusions

The FOCUS measured positive changes in communication skills in all three groups of children after 7–10 h of speech-language therapy. In addition to improvements in specific speech and language skills, the FOCUS measured improved conversation, play and socialization skills. The SI children improved their intelligibility and ability to communicate independently with children and unfamiliar adults. The LI and S/LI children improved their communication effectiveness and social interactions with both children and adults.

An outcome measure that targets only speech and language skills would likely miss the important changes associated with improved communicative participation shown by most of the children. The FOCUS helps clinicians measure the impact of improved communication skills on children's lives. This information will inform clinical practice and increase awareness of the importance of play-based therapy activities in facilitating the child's ability to participation in their world.

**Key messages**

- The FOCUS captures changes in communicative participation for preschool children with a range of communication disorder types and severities.
- An outcome tool that measures only speech and language skills would miss the important changes in communicative participation associated with speech-language therapy.
- Articulation therapy may need to include social skills and play training to help these children gain confidence, enter into play activities and interact better with their peers.
- Further research is needed to determine how communicative participation skills are affected by different treatment approaches.
